# Autoantibody profiles in the sera of patients with Q fever: characterization of antigens by immunofluorescence, immunoblot and sequence analysis

**DOI:** 10.1186/1740-2557-2-10

**Published:** 2005-11-10

**Authors:** MT Camacho, I Outschoorn, A Tellez, J Sequí

**Affiliations:** 1 Departamento de Orientación Diagnóstica. Centro Nacional de Microbiologia. Instituto de Salud Carlos III. Ctra. Majadahonda -Pozuelo Km 12,5. 28080-Madrid. Spain; 2 Departamento de Respuesta Inmune. Centro Nacional de Microbiologia. Instituto de Salud Carlos III. Ctra. Majadahonda -Pozuelo Km 12,5. 28080-Madrid. Spain; 3 Servicio de Inmunología. Hospital Carlos III. Imsalud. c/ Sinesio Delgado n° 10. 28029-Madrid. Spain

**Keywords:** Autoantibodies, *C. burnetii*, Q fever.

## Abstract

Recent reports have shown that some of the immunological aspects of Q fever, a rickettsiosis caused by *Coxiella burnetii*, could be related to self-antigen responses. The aim of this study was to determine the specificity of the autoantibody response of patients with acute and chronic Coxiella infections. Smooth muscle and cardiac muscle-specific autoantibodies were observed in significant percentages in acutely or chronically affected Q fever patients when compared to healthy volunteers. Moreover, the incidence of cardiac muscle-specific autoantibody was significantly higher among chronically ill patients compared to acutely ill patients. Moreover, a band of 50 kD of a HeLa extract was detected in most of the sera of individuals with chronic infections and previous sequence analysis suggests that this antigen presents a high degree of homology with the human actin elongation factor 1 alpha. Further research would be necessary to confirm if antibodies to human cytoskeletal proteins could be of clinical importance in chronically infected Q fever patients.

## Background

Q fever is a worldwide distributed human rickettsiosis that was described by Derrick in 1937. Burnett in Australia and Cox in the United States first isolated its etiological agent almost simultaneously so it was referred to as *Coxiella burnetii *[1,2]. Q fever infection is usually asymptomatic or acute self-limited but Coxiella is an intracellular bacterium that may persist within host macrophages leading to chronic complications such as endocarditis, hepatitis, meningitis, bone infections or chronic fatigue syndrome [3-6]. Diagnosis of Q fever is usually based on serological procedures because isolation of the bacterium is difficult and hazardous and requires level 3 biosafety laboratories [7]. Unique to Coxiella is its antigenic phase variation that appears to involve changes on lipopolyssacharide [2,4,8]. Virulent phase I bacteria are isolated from natural infection while avirulent phase II occurs after serial passages. Although the factors that determine the clinical presentation of Q fever are still not well understood, variation in *C. burnetii *strains, route of infection, host immunity and size of the inoculum have been implicated in disease evolution [2,4,5].

Autoimmune diseases are defined as the pathologic sequelae of autoimmune responses. The precise mechanisms by which they are induced are still under discussion, but genetic, hormonal and environmental factors have all been implicated [9,10]. The idea that infectious agents may represent one of the major environmental factors initiating autoimmune responses is now generally accepted and the mechanisms of induction are currently being re-examined [11-13]. Intracellular microorganisms, particularly those associated with persistent or latent infection, have developed strategies to modulate immune responses and hence survive within host cells [14]. Under these circumstances, these microorganisms would not only initiate but also sustain an anomalous reaction that would lead to the appearance of autoimmune features in predisposed patients. Molecular homology with host sequences, polyclonal stimulation of B and T clones, cytokine stimulation, over expression of major histocompatibility complex molecules (MHC II), antigen modification and host cell damage with the release of self-antigens, are the most commonly described mechanisms that could lead to autoimmune responses due to infectious agents [11,13,15-17]. Links between autoimmunity and infection have been described in several situations, as in the case of myocarditis following coxsackievirus and trypanosomal infections [18-20], rheumatic fever in relation to streptococci [21], ankylosing spondylitis and klebsiella [22], reactive arthropathies and Epstein Barr viruses [23,24] and recently heart disease and chlamydia [25]. Direct evidence that confirms the diagnosis of autoimmune diseases requires test that are still beyond the capacity of most clinical laboratories so autoimmune disease diagnosis is frequently based on circumstantial evidence [26], such as the demonstration and quantitation of the various autoantibodies.

In Q fever patients, the immune responses elicited by *C. burnetii *are associated with inflammatory responses, rheumatoid factor, cryoglobulins or immune-complexes [8,27]. Antibodies to cardiolipin, nuclear antigens, leukocyte, platelet, mitochondrial and smooth muscle antigens are commonly found in chronically infected patients [1,8,27]. The study of IgG subclass distributions has revealed a significant increase in IgG1 and IgG3 levels in sera of patients similarly to those described in some autoimmune diseases [28]. Based on all these findings it could be proposed that some pathological aspects of the long-term chronic Q fever disease, such as heart disease, could be induced or maintained by autoimmune mechanisms. The aim of this study was to characterize the specificity of the autoantigens recognised by circulating antibodies in sera of patients with acute and chronic Coxiella infections and identify differences between chronic diseases with and without cardiac involvement. This may bring new insights to the mechanisms of induction of possible auto-reactive cardiac complications.

## Methods

### Study Subjects

The work was done in accordance with the regulations of internal review board of the Institute of Health, Carlos III. Spain. The patients and volunteers gave a written informed consent on the use of their sera to participate in the study, which was approved by the Human Research Committee.

Serum samples of patients with Q fever. Convalescent sera from 58 serologically confirmed acute (n = 24) and chronic (n = 34) Q fever cases were studied. All patients were adults, equally divided between males and females, aged 26 – 60 years (mean 45 years). Diagnosis was done by immunofluorescence using phase I and phase II Coxiella antigens. A serum sample with phase II IgG titre ≥ 200 and phase II IgM ≥ 50 was considered acute and a serum sample with a phase I IgG titre ≥ 800 was classified as chronic [1,2]. All chronic cases had phase I IgA antibodies titre ≥ 50 [29]. Patients with acute infection had fever and/or atypical pneumonia but those demonstrating serological evidence of chronic Q fever disease could be divided in two groups: those with cardiac involvement (n = 26) or those with other pathologies (hepatic diseases, fever or bone disease) (n = 8).

### Normal control group

Twenty serum samples from healthy volunteers were studied, (males 10, females 10) aged 18 – 36 years (mean 29 years).

### Detection of autoantibodies by indirect immunofluorescence (IIF)

Cryostat sections of monkey cardiac muscle (The Binding Site, UK) were used as a substrate to determine cardiac muscle antibodies (CMA). Sections of rat liver, kidney or stomach (BioSystems, Sp) were used as a substrate to detect specific antibodies (anti-parietal cell, PCA, and anti-smooth muscle, ASMA). Human carcinoma cell line (Hep-2) (MarDx Carlsbad, USA) was used as a substrate to study anti-nuclear antibodies (ANA). Anti-neutrophil antibodies (ANCA) were screened using neutrophil slides (The Binding Site, UK) and anti-double-stranded DNA antibodies (dsDNA) were assayed using *Crithidia luciliae *slides (MarDx Carlsbad, USA).

IIF was done as previously described [30]. Briefly, sera of patients were diluted in phosphate buffered saline (PBS) pH 7.4 (1:40 in ANCA and Hep-2 slides, 1:20 in CMA, dsDNA and rat liver, kidney and stomach slides) and incubated for 30 min at room temperature with each of the sections. Bound antibodies were detected with a fluorescent rabbit anti-human IgG immunoglobulin (Dako, DK). After each incubation, the slides were washed three times for five minutes with PBS. After the final wash, the slides were mounted in 70% glycerol in PBS and examined in a Leitz Diaplan fluorescence microscope. Slides were coded and scored blind by two independent examiners.

### Detection of HeLa antigens specific autoantibodies by Western blot

Assay was carried out using methods previously described [31,32]. Briefly, HeLa cells were cultured at 37°C in 5% CO2 to logarithmic phase in RPMI 1640 supplemented with 10 % heat inactivated fetal calf serum, 1 % glutamin, 10 U/ml penicillin and 60 μg/ml streptomycin. Then, the cells were incubated for 30 min at 4°C in lysis buffer (1 % Nonidet P-40, 150 mM NaCl, 20 mM Tris-HCl pH 8, 1 mM phenylmethylsulfonyl fluoride, 1 μ/ml aprotinin, 1 μ/ml pepstatin, 1 μ/ml leupeptin and 2 mM EDTA). After centrifugation at 20,000 × g for 30 min, the supernatants were stored at -80°C. Protein concentration was determined by BCA assay (Pierce, Pennsylvania, USA). About 400 μg of HeLa whole cell extracts were separated on 12 % SDS-PAGE. Proteins were electrotransferred to polyvinyl difluoride (PVDF) or nitrocellulose membranes in a semi-dry system for 1 h at 5.5 mA/cm2 in carbonate buffer, however ethanol 20 % (v/v) was added instead of methanol. Membranes were soaked for 1 hour in PBS-5 % bovine serum albumin (BSA) and incubated overnight with sera diluted 1:100 in PBS-BSA. After washing, the membranes were incubated for 1 hour with an alkaline phosphatase-conjugated anti-human IgG antibody (The Binding Site. UK) at 1:8000 dilution. After three washes, antigen-bound antibody was visualised with nitroblue tetrazolium and 5-bromo-4chloro-3-indolyl phosphate (Bio-Rad, Richmond, CA) following manufacturer's instructions.

### Protein isolation and N-terminal sequence analysis

HeLa proteins were separated by 8% SDS-PAGE and electrotransferred to PVDF membranes as described above. The membranes were then stained with Coomassie Brilliant Blue R-250 and destained in 50 % methanol. The proteins of interest were cut out from the air-dried membrane and incubated for 30 min with 0.5 % polivinilpirrolidone-40 in 100 mM acetic acid, digested at a enzyme-substrate ratio 1:20 in weight with trypsin in 100 mM sodium bicarbonate at pH 8.2, and then incubated in acetonitrile 95:5 (v/v) for 16 hours at 37°C. After centrifugation at 12.000 × g for 10 min the digestion mixture was acidified with 2 % trifluoroacetic acid and the supernatant injected in a C18 Vydac 2.1 × 250 mm microbore column (Beckman HPLC "System Gold") at a flow rate of 0.15 ml/min in a chromatographic gradient of 7% acetonitrile and 0.09 % of trifluoroacetic acid in water. For peptide micro-sequencing a pulse liquid phase automatic sequencer was used (Applied Biosystems model 473). Sequence homologies obtained with the other proteins were analysed with FASTA and TFASTA programs using the update database from Swissprotein and GenEMBL.

### Data analysis

The results of IIF were analysed using Fisher's exact test whenever appropriate. The overall difference was considered significant when p < 0.05.

## Results

### Detection of autoantibodies by IIF

The substrate specific responses detected in sera of patients with acute and chronic Q fever infections are shown in Table 1.

**Table 1:** Distribution of patients with acute and chronic Q fever that showed specific autoantibodies (IgG isotype) by indirect immunofluorescence.

Specific autoantibodies	Acute Q fever (n = 24)(%)	Chronic Q fever (n = 34)(%)
ANA	3(12.5)	5(14.7)
dsDNA	1(4.1)	3(8.8)
ANCA	3(12.5)	4(11.8)
ASMA	7(29.2)*	9(26.5)*
PCA	0	3(8.8)
CMA	3(12.1)*	13(38.3)#

Total positive patients	13(54.2)	23(67.6)

ANA: Anti-nuclear antibodies;

dsDNA: Anti-double strand DNA antibodies;

ANCA: Anti-neutrophil antibodies;

ASMA: Anti-smooth muscle antibodies;

PCA: Anti-parietal cells antibodies;

CMA: Anti-cardiac muscle antibodies.

*: p < 0.05 as compared with healthy controls

#: p < 0.05 as compared with acute Q fever patients and healthy controls

A total of 54.2% of patients with acute infection had demonstrable responses to at least one of the antigens tested. One patient had antibodies to dsDNA and three had ANCA or ANA but only ASMA and CMA were detectable in a significant number of patients (29.2 % and 12.1% respectively).

**Figure 1:**
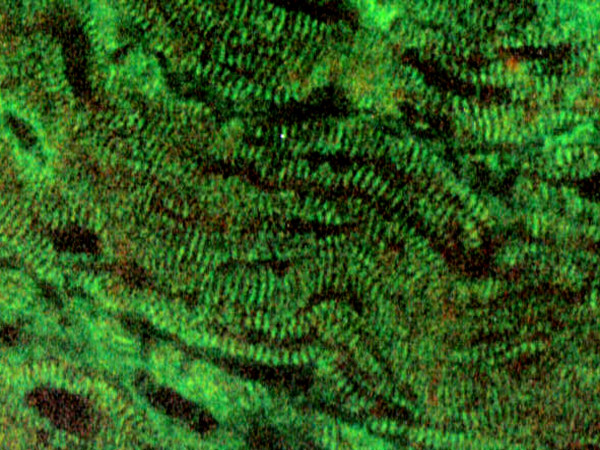
Fibrillar indirect immunofluorescence stain with obtained with sera of Q fever patients using monkey cardiac muscle sections. Magnification × 400.

On the other hand, 67.2% of the patients with chronic Q fever infections showed positive results by IIF, most of them to various antigens tested and no differences were found between patients with or without cardiac involvement. Three patients (8.8%) elicited antibodies to dsDNA or parietal cells, four had ANCA (11.8%) and five ANA (14.7%), and, as in acute patients, the presence of ASMA was detected in a significantly higher percentage of sera (26.5%). CMA were found in 13 patients (38.3%) and both fibrillar and sarcolemma fluorescence stains were found (figure 1). The high frequency of CMA-specific antibody response detected on chronically infected patients was statistically significant (p < 0.05) as compared to patients with acute infection. Sera samples from healthy controls showed no reactivity at the dilutions tested.

### Detection of antibodies by Western blot and sequence analysis

**Figure 2:**
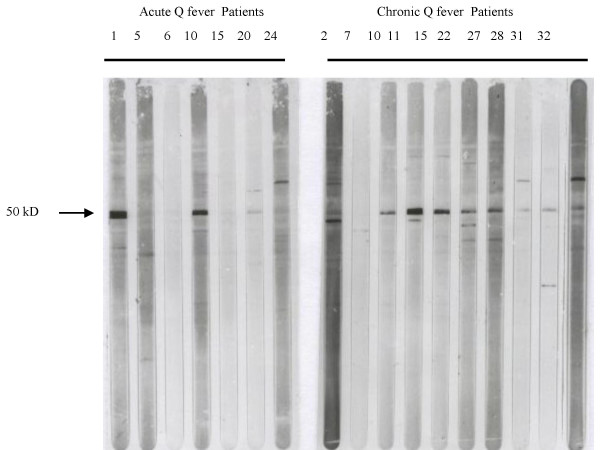
IgG antibodies detected in sera of Q fever patients against partially purified HeLa cell antigens by Western blot analysis. Incubated with sera from acute (number. 1,5,6,10,15,20,24) and chronic (number: 2,7,10,11,15,22,27,28,31,32) Q fever patients. Results obtained with representative sera from each group are depicted. Location of the 50 kD bands are indicated.

**Figure 3:**
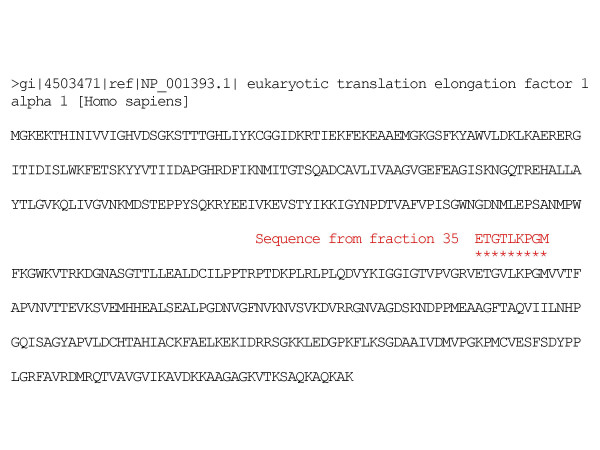
Sequence alignement of a fraction of the chromatogram of the 50 kD HeLa protein trypsin digestion and human elongation factor 1alpha.

Fourteen different HeLa bands, with molecular masses ranging from 20 to 100 kD, were detected by Western blot. Normal sera showed minimal if any reactivity with HeLa proteins. Twelve patients with acute Coxiella infections (57%) showed antibodies that reacted with one to three different bands per sample. By contrast, twenty-five sera of patients with chronic Coxiella infections (75%) showed antibody reactivities to a broad spectrum of up to seven different proteins. A predominant and consistent 50 kD band was observed in 14 of the patients with chronic infections (42%) and in 3 of the acute cases (14%) (Figure 2). No reactivity to this band was found in healthy controls. Sequence analysis of a fragment of this 50 kD protein showed a 98% homology with human elongation factor 1 alpha (Figure 3).

## Discussion

It must be noted that the presence of natural autoantibodies is a normal feature in sera of healthy individuals and their presence may only be circumstantial or transitory. Such natural autoantibodies are usually present at low titre, have poor affinity for their corresponding antigen and usually belong to the IgM class. In autoimmune diseases, self-antigens are recognised by pathological autoantibodies with specificities that differ from those that may occur naturally in healthy humans [26].

As in other infectious diseases, some immunological aspects of the host response to Coxiella in patients with Q fever could be related to self-antigen responses. In our hands, the presence of PCA, ANA, dsDNA and ANCA does not seem to clearly correlate with any of our group of patients and could be only circumstantial. Only ASMA and CMA antibodies were found in a significant percentage of acute and chronically infected Q fever patients as compared to the normal population. In the literature, CMA have rarely been found in normal individuals and their presence has been associated with autoimmune myocarditis, idiopathic dilated cardiomyopathy or rheumatic carditis [33-37]. Many different specificities have been described but mitochondrial and contractile cytoplasmic proteins such as myosin or actin seem to be widely implicated [33,38]. CMA detection following microbial infections has been related to the release of antigens after tissue damage, but in some cases, as in chlamydia infections, Chagas disease, post-streptoccocal rheumatic fever or coxsackie myopericarditis, molecular homologies between host antigens and the etiological agent have been described [11,18,21,25]. CMA presence in the sera of Q fever patients without cardiac involvement was unexpected and suggested that they were not elicited by tissue damage. The possible existence of molecular homologies between coxiella and host antigens was studied by immunoblotting with human HeLa extracts. Results revealed that sera from most of the chronically infected patients and some of acute patients reacted strongly with a 50 kD antigen. Preliminary results of the sequence analysis of an internal fragment of this protein showed molecular homology with human elongation factor 1 alpha. This ubiquitously expressed protein is phylogenetically conserved and an abundant member of the actin binding proteins family, responsible for binding the aminoacyl-tRNA to the ribosome during polypeptide elongation [39-41]. It co-localises intracellularly with the F-actin and is related to changes in the actin cytoskeleton. High levels of its expression are correlated with cell proliferation, oncogenic transformation, metastasis and are been considered a molecular market for injured muscle [42,43] and it has been described as a common IgG auto-antibody target in atopic dermatitis and Felty's syndrome [44,45].

This preliminary study shows that, as in other infectious diseases, patients with Coxiella infections have an autoantibody profile with specificities that resemble those found in autoimmune disease. Further studies would be needed to evaluate whether these findings could only be considered a circumstantial evidence of natural response or have pathological implications.
